# The Timing of Antidepressant Effects: A Comparison of Diverse Pharmacological and Somatic Treatments

**DOI:** 10.3390/ph3010019

**Published:** 2010-01-06

**Authors:** Rodrigo Machado-Vieira, Jacqueline Baumann, Cristina Wheeler-Castillo, David Latov, Ioline D. Henter, Giacomo Salvadore, Carlos A. Zarate

**Affiliations:** Experimental Therapeutics, Mood and Anxiety Disorders Program, National Institute of Mental Health, and Department of Health and Human Services, Bethesda, MD 20892, USA

**Keywords:** antidepressant, depression, ketamine, NMDA, rapid

## Abstract

Currently available antidepressants used to treat major depressive disorder (MDD) unfortunately often take weeks to months to achieve their full effects, commonly resulting in considerable morbidity and increased risk for suicidal behavior. Our lack of understanding of the precise cellular underpinnings of this illness and of the mechanism of action of existing effective pharmacological treatments is a large part of the reason that therapies with a more rapid onset of antidepressant action (ROAA) have not been developed. Other issues that need to be addressed include heterogeneous clinical concepts and statistical models to measure rapid antidepressant effects. This review describes the timing of onset of antidepressant effects for various therapies used to treat MDD. While several agents produce earlier improvement of depressive symptoms (defined as occurring within one week), the response rate associated with such agents can be quite variable. These agents include both currently available antidepressants as well as other pharmacological and non-pharmacological interventions. Considerably fewer treatments are associated with ROAA, defined as occurring within several hours or one day. Treatment strategies for MDD whose sustained antidepressant effects manifest within hours or even a few days would have an enormous impact on public health.

## 1. Introduction 

Major depressive disorder (MDD) is a severe, recurrent, and disabling medical illness, that is highly prevalent worldwide and that is associated with a significant negative impact on productivity and quality of life. In this context, clinical improvement during the first month of treatment with antidepressants is a critical component for achieving long-term stability [1]. However, despite a variety of currently available treatments, many patients do not respond early enough in the course of a major depressive episode. In addition, response is generally considered suboptimal for many of those who do respond. For example, one study of outpatients with MDD found that, despite receiving an adequate trial of a first-line treatment such as a selective serotonin reuptake inhibitor (SSRI), only 29-46% of patients had an adequate response [2]. Similarly, a large multicenter study also found that only a minority of patients with MDD achieved remission within 10-14 weeks [3]. Thus, it is clear that existing antidepressants take considerable time to induce either response or remission. 

Notably, this lag in onset of antidepressant action is associated with negative consequences. Jick and colleagues described an increased risk of suicidal behavior during the first month of antidepressant treatment, particularly during the first nine days; this risk was similar regardless of the chemical class of antidepressant (e.g., amitriptyline, fluoxetine, paroxetine or dothiepin) [4]. It is important to note that higher risk for suicidal behavior and other deliberate self-harm during the first month of treatment may be directly related to increased physical energy in the presence of depressed mood or negative thoughts. Similarly, Simon and colleagues observed a significantly higher risk of suicide attempts during the first week of antidepressant treatment compared to subsequent weeks [5]. Consequently, antidepressants with a more rapid onset of antidepressant effects would be expected to reduce the risk of suicidal behavior [6], and to lead to a more stable long-term response [7,8].

Delayed onset of antidepressant effects can also be associated with psychosocial losses. Depressive episodes limit quality of life by limiting the ability of individuals to function socially and occupationally, thus impairing the skills needed to work, to create and maintain relationships, and to function and be productive across multiple domains [9,10]. Consequences are also associated with ineffective early treatment, including multiple patient visits, long-term psychosocial dysfunction, and consequent lack of adherence. Rapid- or immediate-onset antidepressant effects could also theoretically reduce the harmful neurobiological effects and poor outcomes associated with repeated depressive episodes and enduring depressive symptoms [11]. Thus, as with many other medical disorders, MDD can be considered in many cases an emergency situation that requires immediate intervention to reduce symptom morbidity. 

## 2. The Time Course of Antidepressant Effects

Presently, any discussion of the timing of antidepressant effects associated with either traditional antidepressants or novel therapeutic agents is hampered by the lack of any consistent definition for the terms “early improvement of depressive symptoms” or “rapid onset of antidepressant effects” (ROAA). For the purposes of this review, we define “early improvement of depressive symptoms” as improvement that occurs within one week; however, response rates associated with such agents, including traditional antidepressants from diverse classes, are quite variable (see below). In contrast, agents with ROAA induce significant response rates within a few hours or one day; as we discuss later, considerably fewer agents are associated with ROAA. 

Current definitions of response/remission were developed to detect improvement occurring only after several weeks; thus, whether the same definition could or should apply when defining a response that occurs within a few hours or even a few days is still unclear. In fact, most of the findings regarding the early therapeutic effects of currently available antidepressants were drawn from post-hoc analyses and meta-analyses of clinical trials that were not specifically designed to detect the speed of onset of improvement in depressive symptoms, thus presenting several limitations when attempting to assess this parameter [12].

Treatment response has been widely characterized as a 50% decrease in Hamilton Depression Rating Scale (HAM-D) score compared to baseline. The definition of remission varies considerably from study to study, but at least one widely-used definition describes remission as a HAM-D score lower than 8 [13,14]. In contrast to definitions for response and remission, there is no such general agreement regarding the definition of ‘onset of improvement’. Stassen and colleagues defined ‘onset of improvement’ as the initial moment when HAM-D scores decrease by more than 20% from baseline without a subsequent increase [15]. In that meta-analysis, the estimated rate for onset of improvement (20%) predicted approximately 70% of those who would respond at four weeks. Some guidelines recommend using a 30% change from baseline score to define clinically meaningful improvement [12]. Similarly, Posternak and Zimmerman defined ‘onset of response’ as a sustained reduction of 20 to 33% in global symptom severity [16].

The stringency of current criteria for evaluating rapid antidepressant effects and the lack of standard procedures for measuring it clearly demonstrate the need to assess currently-used depression rating scales according to different validity paradigms potentially associated with an earlier improvement in depressive symptoms [17]. For instance, both further evaluation of the optimal frequency of applying rating scales, and a clear definition of the core symptoms associated with early improvement clearly require further study and the establishment of usable guidelines. Also, criteria for onset of improvement should be directly evaluated in the context of time to improvement, given that a 20% improvement occurring over one to two weeks could be considered less clinically meaningful than the same rate of improvement within one to two hours.

## 3. Pharmacological and Non-Pharmacological Approaches and the Timing of Antidepressant Effects

As noted previously, for the purpose of this review we define ROAA agents as those that induce significant response rates within a few hours or one day of administration. To date, only a few such agents have been definitively identified. They include thyrotropin releasing hormone (TRH), ketamine, and sleep deprivation (SD). Several other agents associated with earlier improvement of depressive symptoms are also being investigated. In this review, we define earlier improvement of depressive symptoms as those that occur within one week, though the response rate associated with such agents can be quite variable. Such agents, reviewed below, include both currently available antidepressants (SSRIs, monoamine oxidase inhibitors (MAOIs), olanzapine-fluoxetine combination (OFC), intravenous administration of standard antidepressants (ISA), and mirtazapine) as well as other pharmacological and non-pharmacological interventions (electroconvulsive therapy (ECT), pindolol, stimulants, hypothalamic pituitary adrenal (HPA) axis inhibitors, repetitive transcranial magnetic stimulation (rTMS), deep brain stimulation (DBS), and the NR2B subunit-selective antagonist (CP-101,606).

### 3.1. The Timing of Onset of Currently Available Antidepressants

Our ability to develop newer, faster-acting, and more effective antidepressants has been hampered by the fact that the biological aspects involved in the first weeks of antidepressant treatment are still poorly understood. In the last few decades, the monoaminergic hypothesis of depression has been considered a useful neurobiological model for explaining the delayed onset of antidepressant response associated with these agents. According to this hypothesis, a reliable and sustained antidepressant response can only occur after two to three weeks of treatment with standard antidepressants [18,19]. Hyman and Nestler further proposed the paradigm of initiation and adaptation to explain the process whereby repeated use of an antidepressant may induce a continuous perturbation of its initial target proteins, subsequently leading to adaptive changes in the neurons controlling the initial target, as well as the adjacent neural circuits [20]. Indeed, converging evidence has shown that the acute pharmacological effects induced by standard antidepressants (during the initial two weeks of treatment) exhibit a different molecular basis than the effects seen during long-term treatment. For instance, increasing intrasynaptic concentrations of serotonin during initial treatment with a SSRI does not immediately lead to an antidepressant response, but chronic treatment does; this effect is presumed to be due to both a subsequent downregulation of the presynaptic serotonergic receptors and increased neuronal firing [21,22]. In this model, the presence of side effects is essential for achieving eventual antidepressant response, a mechanism called tolerance. This model proposes that desensitization of some neurotransmitter receptors is related to the delayed therapeutic action of standard antidepressants, whereas downregulation of other receptors may lead to decreased side effects over time. 

Here, we begin by reviewing studies that more precisely examine the timing of onset of currently available antidepressants, regardless of class. Some studies have suggested that for currently available antidepressants, the average time for onset of antidepressant action is 13 days, but when considering full response criteria, this period goes up to 20 days [15,23]. To study the time necessary for achieving antidepressant response (comparing a true drug effect to placebo response), Quitkin and colleagues used a pattern analysis approach to assess the persistent pattern of response in order to exclude any putative placebo effect [18,19]. Pooling the results of three different trials, they proposed that a real drug-placebo difference could only occur after two to three weeks of treatment, thus emphasizing that true drug-responders would exhibit both delayed onset and sustained antidepressant response, whereas placebo responders would display early but not long-term sustained improvement [18,19]. 

 However, these findings have been questioned by several investigators who demonstrated that early improvement (defined as improvement occurring in the first two weeks) is a real antidepressant effect, and not secondary to placebo response; furthermore, this early improvement potentially predicted subsequent positive long-term outcome. For instance, a recent meta-analysis found that patients using currently available antidepressants had a significantly higher rate of sustained clinical response beginning at Week 1 or 2, compared to individuals receiving placebo [24]. Another study evaluated differences between true antidepressant response and placebo effect in 5,158 patients from 47 studies using a series of sub-analyses. Some patients exhibited a significant difference in active drug versus placebo during the first two weeks of treatment that subsequently persisted at a lower level, thus confirming a clinically representative early distinction between real antidepressant and placebo effects [16]. These data challenge the commonly-held view that standard antidepressants have a delay in onset of action of at least two weeks after treatment begins; indeed, data from a number of recent large-scale studies and meta-analyses suggest that currently available antidepressants can, for some patients, lead to improvement of depressive symptoms within the first week [8,16,24,15,26]. One methodological limitation, however, is that studies investigating an earlier improvement in depressive symptoms assumed that the corresponding response rates were relatively small at this time point. 

Nevertheless, some limitations are present in any discussion of this topic. For instance, when evaluating mean time to response or remission, current antidepressants may show a latency period longer than two weeks. Second, most of the studies pooled in the meta-analyses were not specifically designed to detect rapid antidepressant effects because of the reduced frequency of early assessments as well as the statistical methods used. Finally, available depression rating scales and sub-scales for evaluating early improvement possess limited validity when measuring score changes and differences between real antidepressant and placebo effects for those assessments conducted earlier than a week after treatment onset; obviously, this would affect investigators’ ability to assess improvement that occurs within hours or days. Finally, and as noted previously, the field currently has no clear or standard definition of ROAA compared with “early” or “earlier” improvement of depressive symptoms. 

### 3.2. Differences in Timing of Onset Associated with Antidepressants of Different Classes and Influence on Long-term Outcome

Because depression is a multi-faceted disorder, some investigators have suggested that any measure used to evaluate early improvement should include subscales measuring cognitive, vegetative, and emotional dimensions [12,27,28]. In this context, Katz and colleagues identified eleven depressive constructs using several rating scales during treatment with imipramine and amitriptyline [23,29]. Some of these constructs were shown to significantly change during the first week in patients who responded after four weeks of treatment [29]. Early response to tricyclic antidepressants (TCAs) appeared to be correlated with rapid improvement in measures of anxiety, agitation, hostility, cognitive impairment, depressed mood, and physical expression of distress. Other depressive constructs such as psychomotor retardation showed no early changes. In a subsequent study, the same investigators evaluated possible changes in depressive constructs using antidepressants from two different chemical classes, each primarily affecting a particular neurotransmitter system; desipramine (a noradrenergic reuptake inhibitor) was compared to paroxetine (a SSRI) as well as placebo. Desipramine induced significant early improvement (three to seven days) in psychomotor retardation and depressed mood while paroxetine treatment rapidly improved anxiety. Interestingly, placebo responders showed no specific behavioral pattern [23]. 

This approach has, as yet, not been widely adopted. Only the studies performed by Katz and colleagues have systematically investigated individual depressive dimension changes associated with different antidepressants, or addressed the methodological limitations of previous studies assessing earlier improvement of depressive symptoms (for instance, twice weekly assessments, prospective design, dimensional approaches in symptom analysis, *etc*.). Recently, the same investigators proposed a direct connection between antidepressant-induced neural and behavioral changes (for instance, the link between serotonin and impulsive aggression as well as anxiety, norepinephrine and “arousal” as well as motor activity, *etc.*) and the prediction of clinical outcome based on early response [30]. The authors noted that if only minimal or no antidepressant effects occurred during the first two weeks, non-response at six to eight weeks was the most likely outcome. 

This issue is particularly critical because several studies have demonstrated the influence of early antidepressant effects in predicting long-term outcome [12,28,31,32]. One study that investigated the time course of improvement in patients randomized to different antidepressants (amitriptyline, oxaprotiline, imipramine, moclobemide) found a significant difference between active drug and placebo after five days of treatment, an effect that continued to increase during the first two weeks [33]. Post-hoc analysis showed that early improvement with imipramine or moclobemide during this period strongly predicted response at study endpoint [34]. Another study similarly observed that patients showing a sustained antidepressant response after ten days of treatment were more likely to achieve response after one month [35]. The findings by Katz and colleagues described above suggested that early behavioral changes taking place during the first two weeks of treatment could predict subsequent long-term outcome [23,28,30]. Szegedi and colleagues also noted that early improvement in patients receiving mirtazapine or paroxetine in the first two weeks of treatment predicted response after six weeks of treatment [8]. A similar finding was demonstrated in another study that noted a consistent reduction in depressive symptoms after three days of treatment, and a subsequent distinct course between responders and non-responders at this time point [36]. 

Thus, these findings support the notion that, for some patients, earlier onset of improvement in depressive symptoms can be associated with particular antidepressants and that this earlier response may correlate with better short- and long-term outcome. The development of homogeneous study design and terminology, as well as the implementation of standard statistical analyses related to measures of time course of improvement are necessary to increase the validity of these findings. Below, we review some of the most salient findings regarding specific antidepressants and the timing of their ability to resolve depressive symptoms.

#### 3.2.1. SSRIs

Several studies have examined whether citalopram and its enantiomer escitalopram can achieve an earlier onset of antidepressant action than other standard antidepressants [37,38]. Patients receiving citalopram had significantly decreased HAM-D and Montgomery-Asberg Depression Rating Scale (MADRS) scores at Week 2 compared to patients receiving fluoxetine or sertraline [39,40]. Moreover, a recent pooled analysis of eight clinical trials in MDD found that escitalopram induced greater improvement in MADRS scores at Week 1 than placebo, other SSRIs, or venlafaxine [37]. 

In addition, the adjunctive use of tryptophan to fluoxetine was noted to result in an earlier onset of antidepressant effect and protective profile on slow-wave sleep [41]. However, no follow-up studies were conducted after this initial finding. 

#### 3.2.2. Monoamine Oxidase Inhibitors (MAOIs)

MAOIs have demonstrated similar or higher rates of response compared to antidepressants of other classes [42,43]. For instance, clinical improvement at Week 2 was shown with phenelzine, which correlated with rate of MAO inhibition [44]. One double-blind study observed superior response rates and time to response using MAOIs in atypical depression compared to imipramine or placebo, with additional superiority after six weeks of treatment [45]. Amsterdam and Shults suggested that MAOIs may be superior to other classes of standard antidepressants [46]. Supporting this notion, a crossover study evaluating the MAOI tranylcypromine in subjects with bipolar depression who were non-responsive to imipramine found that 75% responded to tranylcypromine [47]. However, caveats of using MAOIs include the dietary requirements necessary with their use and their side effect profile. 

#### 3.2.3. Olanzapine-Fluoxetine Combination (OFC)

The combination of fluoxetine and the atypical antipsychotic olanzapine has proven to be useful for achieving antidepressant response in both MDD and bipolar depression [48,49,50]. A large, multi-center, double-blind study evaluated the efficacy of OFC (n = 146), olanzapine (n = 144), fluoxetine (n = 142), or nortriptyline (n = 68) in treatment-resistant MDD [50]. A significant improvement in depressive symptoms was observed with OFC (as assessed by MADRS scores) compared to nortriptyline from the first week of treatment. OFC was also associated with earlier onset of action, as early as Week 2 compared to the other groups. Notably, these differences tended to decrease over the course of the trial; by endpoint, there was no significant difference between OFC and other antidepressant treatments in terms of change from baseline, or response and remission rates [50]. A similarly designed study that included patients with treatment-resistant MDD found a significant decrease in MADRS scores from Weeks 1 through 6 in the OFC group compared to patients using monotherapy with olanzapine, fluoxetine, or venlafaxine [49]. Another study that examined the effects of monotherapy olanzapine augmentation to standard antidepressants found that this combination was not associated with earlier onset of antidepressant action compared to placebo; the negative results could partly be due to the small sample size [51]. Taken together, these results suggest that OFC treatment may yield earlier onset of antidepressant action than other traditional antidepressants. 

#### 3.2.4. Intravenous Standard Antidepressants (ISA)

Anecdotal data have described earlier onset of antidepressant effects using ISA compared to standard administration; these effects are believed to be due to ISA’s superior plasma concentrations and long-lasting bioavailability [52]. For instance, the IV use of the TCA clomipramine produced significant antidepressant effects as early as the first week of treatment [53,54]. However, randomized controlled trials failed to demonstrate superior efficacy or earlier onset of action for IV clomipramine compared to the oral compound [55,26,57]. After seven days of treatment, the high dose of IV amitryptiline (150 mg/day) initially showed superiority over the same oral dose; however, no difference in number of responders was observed after 14 days of treatment [58]. Similarly, IV administration of the SSRI citalopram showed no advantage over the oral preparation in two double-blind studies [59,60]. Taken together, these findings do not support ISA as having an earlier onset of antidepressant action than oral antidepressants. 

#### 3.2.5. Mirtazapine

Mirtazapine is an α_2_ adrenoreceptor antagonist that enhances both serotonin and norepinephrine transmission. Preliminary studies using mirtazapine found a significantly earlier onset of action compared to various SSRIs, including fluoxetine, paroxetine, citalopram and sertraline (reviewed in [22]). Similarly, Quitkin and colleagues described its increased efficacy compared to fluoxetine, citalopram, and paroxetine during the first week of treatment (13% *vs*. 6%) [61]. Recent studies comparing the clinical efficacy of mirtazapine and venlafaxine found a similar global efficacy, but a consistently faster onset of action with mirtazapine. Mirtazapine significantly improved psychomotor retardation, sleep, anxiety, and the Bech-6 factor sub-item compared to venlafaxine at several time points within the first two weeks of the study [25]. In contrast, a randomized trial failed to show any superiority for mirtazapine over fluoxetine in terms of early onset of action or at endpoint [62]. Overall, these data suggest that there is some evidence that mirtazapine may yield earlier antidepressant effects than other standard antidepressants, a benefit that cannot be entirely accounted for by its sedating properties [22,25].

### 3.3. Other Pharmacological and Somatic Interventions Currently Being Investigated for Earlier Antidepressant Effects

#### 3.3.1. Electroconvulsive Therapy (ECT)

ECT has been considered the most effective and rapid-acting long-term somatic treatment in psychiatry [63]. Some studies found an earlier onset of antidepressant response with ECT compared to imipramine and paroxetine [64,65]. Other studies also described significant improvement in HAM-D scores after a single ECT application [66,67]. Earlier onset of antidepressant response induced by ECT was also shown to correlate with better long-term outcome [68]. In a cohort of 253 depressed patients receiving ECT three times a week, Husain and colleagues observed that more than 50% achieved response within the first week, and 83.4% responded within two weeks [69]. This same study also described a sustained response in 34.8% of patients during the first week and in 64.4% within the second week (at or before their sixth session of ECT); 34% experienced remission before or at the end of Week 2. Taken together, these findings suggest that an earlier improvement in depressive symptoms can be achieved with ECT for a large percentage of patients. 

#### 3.3.2. Pindolol

Augmenting serotonergic central metabolism is believed to play a central role in the therapeutic effects of diverse antidepressants [70]. As noted above, current theories regarding the latency of antidepressant effects are based on preclinical findings demonstrating that exposure to SSRIs initially reduces serotonergic firing on interneurons, mostly due to overstimulation of the inhibitory 5HT_1A_ autoreceptors in the dorsal raphe nucleus subsequent to an initial increase in serotonin levels [71]. A PET study found that at a dose of 5 mg t.i.d., the 5-HT_1a_ blocker pindolol led to only a modest (19%) occupancy of the 5-HT_1a_ autoreceptor [72]. In healthy subjects, pindolol induced significant REM sleep suppression with posterior REM rebound after withdrawal [73], suggesting that the manipulation of 5-HT_1A_ autoreceptors directly influences brain serotonergic function.

Since the first placebo-controlled trial showing a faster onset of antidepressant action using pindolol as an add-on treatment to SSRIs, more than fifteen trials have been performed [74]. A recent meta-analysis evaluated both early and late outcomes with pindolol augmentation; while a consistent superiority of pindolol over placebo was demonstrated for early outcome, a combined analysis evaluating longer-term efficacy (four to six weeks) found no significant difference between groups [75]. In addition, pindolol failed to show efficacy in treatment-resistant MDD [74,76,77]. Some studies have also described earlier improvement of depressive symptoms using pindolol based on its ability to rapidly desensitize 5-HT_1A_ autoreceptors and regulate serotonergic neuronal firing rates [78]. Interestingly, it has been proposed that early inhibition of serotonergic reuptake on its projection areas is critically relevant for achieving earlier improvement of depressive symptoms with pindolol treatment. These findings suggest that the earlier improvement of depressive symptoms induced by pindolol augmentation to SSRIs seems to be only limited to the first two weeks of treatment in non-refractory depression, whereas after this period SSRIs are able to desensitize the pre-synaptic autoreceptors, thus bringing about their own therapeutic effects.

#### 3.3.3. Stimulants

Anecdotal data suggest that stimulants may shorten the latency period for antidepressant response. For instance, early reports noted an earlier onset of antidepressant effects with the stimulant benzedrine [79]. Methylphenidate was found to hasten antidepressant response when added to TCAs in an open label study (30% and 63% response after one and two weeks of stimulant treatment, respectively), especially in depressed patients without significant anxiety [80]. However, two controlled studies failed to confirm that stimulants were associated with earlier antidepressant benefits. A double-blind, placebo-controlled study failed to show an earlier onset of antidepressant effects using methylphenidate as add-on therapy to sertraline [81], and a subsequent study also found no difference between placebo and adjunctive methylphenidate for treatment-resistant MDD [82]. While it is potentially possible that some stimulants may be able to induce earlier onset of antidepressant effects due to their dopaminergic properties, this class of agents should be used with caution in patients whose MDD is associated with psychotic features or who have bipolar depression. 

#### 3.3.4. Hypothalamic Pituitary Adrenal (HPA) Axis Inhibitors

In recent years, several drugs that target the HPA axis have been investigated for their potential antidepressant properties. Consistent evidence exists that both MDD and bipolar disorder are associated with HPA axis dysfunction. Specifically, depressed patients show increased levels of corticotropin-releasing hormone (CRH), adrenocorticotropic hormone (ACTH), and cortisol compared to normal controls, which purportedly normalize after antidepressant response. CRH receptor antagonists, glucocorticoid receptor (GR) antagonists, and steroid synthesis inhibitors are the three major classes of these compounds being investigated in the treatment of mood disorders. 

The non-selective GR antagonist mifepristone (RU-486) was associated with antidepressant and antipsychotic effects in psychotic depression [83]. In a six-week trial in individuals with bipolar depression, mifepristone (600 mg/day) improved depressive symptoms and cognitive functioning compared to placebo [84]. However, two large Phase III studies of individuals with psychotic depression found no such antidepressant efficacy [85]. Future studies are needed to clarify whether mifepristone elicits a different response in MDD with psychotic features versus bipolar depression. It is important to note that long-term treatment would be associated with significant side effects (e.g., adrenal insufficiency, hepatic injury, antiprogesterone effects [86,87]), thereby limiting its use to acute depressive episodes.

In preclinical studies, altered CRF1 receptor activity critically regulated depressive-like behaviors. As a result, diverse nonpeptide CRF1 receptor antagonists have also recently been tested preclinically as potential antidepressant agents (reviewed in [88]), including antalarmin [89], the CRF inhibitor CP-154,526, and the 2-aminothiazole derivative SSR125543A [90,91,92]. In humans, an open label study using the CRF1 antagonist R-121919 found that it decreased anxiety and depressive symptoms in patients with MDD [93].

Another recent, double-blind, placebo-controlled trial investigated the antidepressant effects of add-on treatment (nefazadone or fluvoxamine) with metyrapone, a steroid synthesis inhibitor, in hastening antidepressant response [94]. Survival analysis showed a significantly earlier onset of antidepressant action (as assessed by HAM-D scores) in the group receiving adjunctive metyrapone as early as Week 1. Metyrapone was also more effective than placebo (added to other antidepressants) in achieving response at Day 21 and Day 35, while the remission rates at Week 5 were comparable between the two groups. Though these data are encouraging, the possible clinical usefulness of HPA axis modulators in obtaining an earlier onset of antidepressant action needs further investigation. It should also be noted that chronic use of HPA axis inhibitors could lead to hyperandrogenism and hyporcortisolism.

#### 3.3.5. Repetitive Transcranial Magnetic Stimulation (rTMS)

rTMS is a non-invasive tool that has recently been used to treat MDD. The precise biological mechanisms involved in its mechanism of antidepressant action have yet to be elucidated [95,96]. Rumi and colleagues observed a significantly faster antidepressant effect during the first week of treatment when rTMS was used to augment amitriptyline compared to sham rTMS (placebo) plus amitriptyline [97]. Another placebo-controlled study also found faster antidepressant onset (Week 1) using rTMS (in the dorsolateral prefrontal cortex) as an add-on treatment to venlafaxine, escitalopram, or sertraline [98]. Overall, the role of rTMS in achieving earlier improvement of depressive symptoms is promising but needs to be confirmed in large, double-blind, controlled studies.

#### 3.3.6. Deep Brain Stimulation (DBS)

DBS is a surgical treatment involving the implantation of a medical device that sends electrical impulses to specific parts of the brain. It is approved as a treatment for Parkinson’s disease and dystonia, and is also routinely used to treat chronic pain. In patients with treatment-resistant MDD, DBS reduced elevated activity in the subgenual cingulated cortex (Cg25). Although clinical response was not directly assessed, symptoms of depression were noted to improve within a short period of time [99]. After an initial report describing six cases, fourteen more patients with MDD were included in a DBS trial. DBS produced progressive improvement of depressive symptoms over time; specifically, antidepressant response was seen in 40% of patients one week after surgery, in 60% of patients at six-month follow-up, and in 55% of patients at one-year follow-up [100]. Placebo-controlled trials are currently underway; if clinical efficacy is confirmed, DBS may become a valid therapeutic option for some patients with MDD.

#### 3.3.7. NR2B Antagonists

Following the promising findings obtained with ketamine (see below), the potential rapid antidepressant efficacy of the NR2B subunit-selective antagonist CP-101,606 was investigated [101]. The study evaluated paroxetine nonresponders who were subsequently randomized to receive either a single, double-blind IV infusion (0.75mg/kg in 1½ h) of either CP-101,606 or placebo (1:1) adjunctively to paroxetine 40mg/day. Sixty percent of the treatment-resistant patients with MDD using the active compound met criteria for response (50% improvement in HAM-D scores) and 33% achieved remission at Day 5. Among responders at Day 5, 32% showed response one month after infusion. Despite the promising results, the study was associated with some limitations. These include that the study compound was not tested as monotherapy, that paroxetine also affects the glutamate system, and that the study blind may have been compromised due to the dissociative side effects associated with the study compound and subsequent dose modification (both total dose and duration of the infusion). 

### 3.4. Interventions Associated with ROAA

#### 3.4.1. Thyrotropin Releasing Hormone (TRH)

The tripeptide TRH modulates serotonergic, dopaminergic, and glutamatergic transmission in cortical and limbic areas [102,103,104]. Two separate studies noted antidepressant effects occurring within hours after intravenous administration of a single dose of TRH in subjects with MDD, an improvement that persisted for three days [105,106]. However, other studies failed to replicate these initial findings [107,108,109]. The negative results may be due to use of a low TRH dose or intravenous route of administration instead of intrathecal; the former has been associated with very rapid enzymatic degradation. To address this latter methodological issue, Marangell and colleagues administered intrathecal TRH to eight patients with treatment-resistant MDD and measured the onset of antidepressant effects using an abbreviated version of the HAM-D [110]. Five of eight patients showed antidepressant response (within 32 hours) that lasted one to two days. Interestingly, TRH was also associated with a rapid decrease of suicidality. 

In addition to route of TRH administration, the timing of TRH administration also seems to be a key issue in studying its antidepressant properties. For instance, nocturnal intravenous TRH induced rapid clinical response within 24 hours of administration [111] in patients with bipolar depression. There are, however, limitations associated with these studies, including lack of replication and the imbalance of thyroid-stimulating hormone (TSH) levels between groups. Very little research in this area has been conducted in recent years; further placebo-controlled studies with larger sample sizes and clear evaluation of TRH kinetics and bioavailability are necessary requisites for clarifying TRH’s possible ROAA. 

#### 3.4.2. Ketamine

Recently, Zarate and colleagues showed that a single infusion with the *N*-methyl-D-aspartate (NMDA) antagonist ketamine induced a rapid (within two hours) and sustained (one to two weeks) antidepressant effect in patients with treatment-resistant MDD [112]. Previous work by Berman and colleagues had described rapid antidepressant effects with ketamine in seven patients [113]. The randomized, double-blind, placebo-controlled, crossover study by Zarate and colleagues used a single intravenous subanesthetic dose of ketamine (0.5 mg/kg for 40 minutes); 71% of patients experienced a significant antidepressant response within 24 hours after a single dose of ketamine, and this response was sustained for more than one week [112]. More than 70% of patients met the criteria for response (50% improvement) at 24 hours after infusion, and 35% presented a sustained response after one week. Two patients maintained their response for at least two weeks in the ketamine group, and no patient responded in the placebo group. Patients were rated 60 minutes prior to infusion and at 40, 80, 110, and 230 minutes, as well as one, two, three, and seven days after the single intravenous dose. Significant improvement in the 21-item HAM-D with ketamine over placebo was continuously observed from 110 minutes through seven days. The effect size for the drug difference was very large (d = 1.46; 95% CI, 0.91–2.01) after 24 hours, and moderate to large (d = 0.68; 95% CI, 0.13–1.23) after one week. Similar improvements were noted using the Beck Depression Inventory and Visual Analogue Scale depression scores. The core symptoms of MDD were significantly attenuated or, in many cases, completed remitted in the first few hours following ketamine infusion. Mild perceptual disturbances were observed in most patients only in the first hour after infusion; no serious adverse events occurred.

Interestingly, ketamine also significantly reduced suicidal ideation in individuals with treatment-resistant MDD [114,115]. The ROAA associated with a single infusion of ketamine is believed to be secondary to increased glutamatergic throughput of AMPA relative to NMDA receptor [116]. In addition, the relatively sustained antidepressant effects (lasting approximately one to two weeks) resulting from a single intravenous infusion of ketamine are presumed to be due to early plasticity changes in critical local neuronal circuits involved in mood and behavior [112,117,118]. Ketamine has also been found to have antidepressant effects in animal models of depression [119,120], including those in which animals received only a single dose of ketamine [116]. 

#### 3.4.3. Sleep Deprivation

Sleep deprivation (SD) has consistently been shown to induce rapid, dramatic, but transitory antidepressant effects in depressed patients that usually last until recovery sleep takes place [121]. Studies have suggested a direct role for serotonergic and noradrenergic neurotransmission in the ROAA of SD. The serotonin-mediated effects have been shown to decrease the sensitivity of 5-HT_1A_ autoreceptors after total SD [122]. Recent genomic and proteomic studies have described that, similar to treatment with antidepressants, SD rapidly up-regulates different plasticity-related genes such as cAMP response element binding (CREB) and brain derived neurotrophic factor (BDNF); these genes have been reported to be the common final targets of existing antidepressants. 

Interestingly, the potential activation of plasticity-induced pathways during SD appears to be predominantly mediated by the activation of noradrenergic projections in the locus coeruleus. One night of SD has been demonstrated to stimulate hippocampal neurogenesis, but opposite effects have also been described [123,124]. Pharmacological activation of the noradrenergic system during REM could generate an antidepressant effect by a mechanism similar to SD, allowing an interaction with a sensitized postsynaptic milieu, thus rapidly and directly increasing the expression of neuroplasticity genes rather than delaying the indirect effect as occurs with existing antidepressant treatments [125]. 

High rates of relapse (around 80%) are common following sleep recovery [121,126]. Thus, it has been suggested that the response to SD is only transient and does not represent a “real” sustained antidepressant response. Given the highly consistent initial, but ultimately unsustainable, rapid antidepressant effects of SD, several different pharmacological and non-pharmacological strategies have been attempted to maintain this ROAA. Interestingly, medicated patients undergoing SD seem to show significantly lower relapse rates compared to drug-free subjects [121]. Some studies have noted that acute and chronic adjunctive treatment with lithium significantly augmented and sustained the rapid improvement associated with partial or total SD [127,128,129]. Studies evaluating the potential ROAA of SD augmented by pindolol, light therapy, or sleep synchronicity have also been conducted, but need further replication [130,131,132]. Another study found that adding three noninvasive circadian-related interventions to SD in medicated patients boosted and maintained antidepressant response in individuals with bipolar disorder [133]. Recently, the ROAA associated with SD was found to correlate with serum BDNF changes in MDD [134]. 

This model seems to be useful and reliable for studying specific validity paradigms related to the neurobiological basis of depressive episodes, but its putative long-term antidepressant effects—either as monotherapy or as an add-on strategy—have not yet been demonstrated. 

## 4. Future Directions 

Currently available antidepressant medications all exhibit a delayed onset of antidepressant response, resulting in considerable morbidity, disruption to personal, professional, family, and social life, and elevated suicide risk. Any antidepressant treatment that shifts the time frame of response from weeks to a few hours would undoubtedly revolutionize the care of the millions of individuals who suffer from MDD. 

Most current approaches for examining early or rapid improvement in depressive symptoms have focused on weekly evaluation. As a result, the field of psychiatry lacks any consistent definition of “earlier” improvement in depressive symptoms or ROAA. In this review, we have defined the former as occurring within one week of administration, and the latter as occurring within hours or one day of administration. In this respect, the recent studies conducted with sleep deprivation, ketamine, and TRH provide direct evidence that ROAA in a few hours is possible. Therefore, any new paradigm to evaluate ROAA must include the evaluation of antidepressant efficacy occurring within hours or days of first administration. In this context, future studies focusing on the onset of antidepressant effects should address three major issues: 1) the time necessary for antidepressants to induce a significantly greater therapeutic effect (in overall symptoms) compared to placebo, and the potential association of this effect with other outcomes such as response and remission; 2) the timing of improvement in individual depressive symptoms and constructs, given that some specific symptoms or groups of symptoms (clusters) may remit faster than others depending on the class of antidepressant used; and 3) elucidating the relationship between early improvement and long-term effect, which is critical for confirming any agent’s potential ability to affect long-term outcome. Also, it should be pointed out that many substances are capable of inducing transitory euphoria and hyperactivity limited to the half-life of the compound administered, but these effects cannot be characterized as improving core depressive symptoms. 

As the search for treatments for MDD continues, it is crucial to change the way we understand and conduct drug development. As with other areas of medicine, our gradual understanding of the pathophysiology of MDD and the mechanism of action of currently available antidepressants indicates that an antidepressant response that occurs within hours is now an obtainable goal. As this review has highlighted, there is direct evidence that such a rapid response is possible. Therefore, instead of developing treatments that take weeks to induce response, the next generation of antidepressants should aim to resolve symptoms within hours, thereby alleviating much of the suffering associated with MDD.
